# Verification of successful maintenance by serum drug level during a guided antipsychotic reduction to reach minimum effective dose (GARMED) trial

**DOI:** 10.1017/S0033291724002356

**Published:** 2024-10

**Authors:** Chun-I. Liu, Chih-Min Liu, Huai-Hsuan Chiu, Chia-Chi Chuang, Tzung-Jeng Hwang, Ming H. Hsieh, Yi-Ling Chien, Yi-Ting Lin, Ko Yen, Chen-Chung Liu

**Affiliations:** 1 Department of Psychiatry, National Taiwan University Hospital, Taipei, Taiwan; 2 Department of Psychiatry, College of Medicine, National Taiwan University, Taipei, Taiwan; 3 Department of Medical Research, National Taiwan University Hospital, Taipei, Taiwan

**Keywords:** aripiprazole, concentration, dose tapering, minimum effective dose, schizophrenia, therapeutic drug monitoring

## Abstract

**Background:**

Inconsistent results regarding the risk of relapse and better subjective outcomes of previous antipsychotic dose reduction trials in patients with remitted psychosis have not been verified using therapeutic drug monitoring (TDM). This study examined plasma drug concentrations of a dose-tapering trial which exhibited the potential of successful maintenance under lower antipsychotic dosages.

**Methods:**

A 2-year open-label randomized prospective trial recruited remitted patients to undergo guided antipsychotic tapering. Blood samples were collected at baseline, annually, and after each dose reduction. Plasma aripiprazole/dehydroaripiprazole concentrations were determined using LC–MS/MS. The relationship between the dose and serum drug levels was examined using Spearman's correlation. Divided at 120 ng/mL, relapse rate, global function, quality of life, and psychopathology were compared between high- and low- drug level groups.

**Results:**

A total of 126 blood samples were collected, after excluding13 samples due of non-adherence. The correlation coefficients between dosage and drug level were 0.853 (aripiprazole) and 0.864 (dehydroaripiprazole), and the dose and concentration plots were parallel along the tapering trajectories, except patients with non-adherence. The concentration-to-dose ratio of aripiprazole in this cohort, 17.79 ± 7.23 ng/mL/mg, was higher than that in Caucasian populations. No significant differences were observed in the clinical outcomes between the high- and low-level groups. Remarkably, 12 of 15 patients maintained remission at plasma aripiprazole concentrations of <120 ng/mL.

**Conclusions:**

The lower-than-expected doses reached in our antipsychotic tapering trial were substantiated to provide adequate prophylactic effects by TDM results in a subset of patients treated with aripiprazole, even considering the differences in pharmacogenomics between ethnicities.

## Introduction

Antipsychotic medications are considered the primary choice for alleviating psychotic symptoms and cognitive deficits for patients with psychotic disorders (Bowie et al., 2018; Sheffield, Karcher, & Barch, 2018). Prior research has consistently demonstrated that discontinuation of antipsychotics is associated with a higher risk of relapse (Dunayevich et al., 2007; Kishi et al., 2019; Üçok & Kara, 2020). In addition, the discontinuation of antipsychotic medication is linked to higher rates of inpatient hospitalization, increased emergency department visits, and a greater incidence of somatic adverse events, resulting in higher healthcare costs (Bai et al., 2017; Brandt et al., 2022; Chou et al., 2017; Iwamoto et al., 2012; Joo et al., 2022; Khandker, Chekani, Limone, & Riehle, 2023; Ostuzzi et al., 2022; Rodolico et al., 2022; Sabé et al., 2023; Sommer, Horowitz, Allott, Speyer, & Begemann, 2022; Sumiyoshi, 2008). Dose reduction might not be a feasible solution, as a matched cohort study revealed an increased risk of emergency room and inpatient visits, relapses, and the diagnosis of tardive dyskinesia (Zichlin, Mu, Leo, & Ayyagari, 2021). Intermittent treatment is not as effective as maintenance treatment, although it is better than no antipsychotic treatment (Sampson, Joshi, Mansour, & Adams, 2013).

However, a previous study revealed a U-shaped relationship between overall mortality and cumulative exposure to antipsychotics (Torniainen et al., 2015). Further investigations concluded that the experience of dose reduction is influenced by diverse factors, such as variations in dose reduction profiles, effects of reduction, individual motivation and engagement levels, and the nature of relationships with prescribers (Jauhar, Fusar-Poli, & Foreman, 2023; Morant et al., 2023). Additionally, medication tapering has been proposed as a potential approach to facilitate functional recovery in individuals with psychotic disorders (Lahera et al., 2016; Singh et al., 2022). A more individualized and slower tapering strategy should be applied based on patient characteristics to optimize the balance between relapse rate and well-being (Barnes et al., 2020; Liu & Takeuchi, 2020; McCutcheon et al., 2024; Sommer et al., 2022; Wunderink, 2019).

Currently, efforts are being made to address evidence-to-practice gaps regarding dose tapering in practical scenarios (Koops et al., 2024). A small-sample randomized controlled pilot trial showed no significant increase of relapse rate in the dose reduction group within 26 weeks (Huhn et al., 2021), whereas an open-label study (the RADAR trial) comparing dose reduction to maintenance treatment failed to identify the benefits of dose reduction at the expense of a higher readmission rate by the end of 2 years (Moncrieff et al., 2023). These contradictory findings, together with those of previous dose discontinuation trials (Gaebel, Stricker, & Riesbeck, 2020; Wunderink, Nieboer, Wiersma, Sytema, & Nienhuis, 2013), indicate that none of them employed therapeutic drug monitoring (TDM) to ascertain the dose taken by patients. It is worth noting that if patients who experience relapse during tapering stop antipsychotics faster than the recommended tempo or even discontinued abruptly, such a relapse should not be attributable to failure of the protective effect under a lower dose of antipsychotics. Conversely, those who remained well during the tapering process might not have reduced the designated dose but maintained it at a dose they felt comfortable with. Thus, we face a pivotal challenge regarding the validity and reliability of the relationship between antipsychotic use and prophylactic effects during remitted states.

The use of TDM as a problem-solving tool in antipsychotic treatment was advocated by a joint expert consensus statement (Schoretsanitis et al., 2020). To date, various studies have endeavored to establish empirical therapeutic ranges of antipsychotics (Grundmann, Kacirova, & Urinovska, 2014; Urban & Cubała, 2017). Specific to aripiprazole, Hart et al.'s comprehensive analysis, which synthesized findings from previous studies, suggested a therapeutic range of 120–270 ng/mL of serum aripiprazole level with an initial dose of aripiprazole of 10 mg/d and 5 mg/d for poor metabolizers. Therefore, the threshold for therapeutic efficacy is set at a serum level of 120 ng/mL.

Recently, we demonstrated the feasibility of a guided antipsychotic reduction project to reach minimum effective dose (GARMED trial) (Liu et al., 2022, 2023b). The procedure is similar to that proposed by Horowitz et al., considering the D2 blockade dynamics (Costardi, Gadelha, & Bressan, 2021; Horowitz, Jauhar, Natesan, Murray, & Taylor, 2021a; Horowitz, Murray, & Taylor, 2021b). Participants were categorized into three groups: gradual dose reduction, controlled dose maintenance, and natural dose maintenance. Guided by an exponential tapering strategy with a 24-week reduction interval repeated in 2 years, the gradual dose reduction group successfully achieved a lower dosage (on average reduced 41% of baseline doses) and better outcomes without an increased risk of relapse compared to two counterparts (Liu et al., 2023b). In this study, we aimed to (1) verify the reported dosage in the GARMED trial by examining the serum drug levels of aripiprazole and its active metabolite, dehydroaripiprazole, at different doses; (2) compare the aripiprazole concentration-to-dose ratio of this population to previous studies; and (3) compare the clinical features and outcomes between participants with high and low serum drug levels.

## Methods

### Study design and setting

The present study was an open-label, randomized, prospective, comparative cohort investigation involving patients diagnosed with schizophrenia-related psychotic disorders based on the Diagnostic and Statistical Manual of Mental Disorders, Fifth Edition [DSM-5]. The study protocol has been previously published (Liu et al., 2022) and is briefly described below. All participants were recruited by investigators from the psychiatric outpatient and daycare services of National Taiwan University Hospital in Taipei, Taiwan. The study was conducted between August 2017 and September 2022.

Prior to participating in the trial, all participants were informed of the risks and benefits associated with long-term antipsychotic treatment and the rationale behind the trial. The participants provided written informed consent to participate in the study, which was conducted in accordance with the principles outlined in the Declaration of Helsinki and International Conference on Harmonization Good Clinical Practice guidelines. The study protocol and any subsequent amendments were approved by the Research Ethics Committee of National Taiwan University Hospital (REC: 201703002RIND) and the trial was registered with ClinicalTrials.gov (identifier: NCT03248180).

### Participants

Eligible participants were in remitted states of psychosis while maintaining a fixed dose of antipsychotic medication for at least 3 months prior to recruitment. Patients with unstable symptoms (a score of ⩾5 on any of the 30 Positive and Negative Syndrome Scale (PANSS) rating items or a score of ⩾4 on any of the 5 PANSS items, P1: delusion, P2: conceptual disorganization, P3: hallucination, G9: unusual thought, G5: mannerism and posturing), admission to an acute psychiatric ward within the past 6 months, concurrent use of mood stabilizers, revising doses of concomitant psychotropic agents within the previous 3 months, a history of substance dependence within the past 6 months, or current pregnancy or breastfeeding status were excluded.

### Patient grouping and dose tapering

Eligible patients who agreed to taper antipsychotic doses were randomly assigned in a 2:1 ratio to either the exponential drug reduction (GDR) or maintenance treatment group (MT1). Eligible patients who declined to try tapering yet agreed to receive follow-up were designated as the naturalistic comparison group (MT2), in which they would continue to maintain their antipsychotic dosage.

In the GDR group, participants underwent an exponential gradual reduction in antipsychotics, beginning with a maximum reduction of 25% of their baseline doses. Subsequently, the patients underwent close monitoring and regular assessments. The next dose reduction, employing an exponential model to reduce an additional 25% of the current dosage (reaching 56.25% of the baseline dose), was not initiated until a 6-month of stabilization was achieved. At each time point eligible for the next tapering, the participants were asked to decide whether to further reduce their dosage or maintain the current level. This dose reduction schedule was repeated at 6-month intervals throughout the 2-year follow-up period. The rationale for using such an innovative dose reduction algorithm has been provided in a previous publication (Liu & Takeuchi, 2020).

### Therapeutic drug level measurement

#### Blood sample collection and storage

Blood samples were collected from all participants to measure the aripiprazole and dehydroaripiprazole concentrations at three time points: baseline, 1-year follow-up, and 2-year follow-up. In the GDR group, samples were collected every 6 months after treatment with a stable dose (prior to the next tapering) or 1 month after any dose adjustment. Blood samples were centrifuged at 3000 rcf at 4 °C for 15 min to obtain serum samples, which were then stored and frozen at −80 °C before use.

#### Sample preparation for aripiprazole and dehydroaripiprazole measurement

Plasma samples (100 μL) underwent extraction with 400 μL of extraction solution composed of 10% formic acid in a 1:1 mixture of acetonitrile and methanol, followed by a 2-min Geno/Grinder2010 extraction step performed at 1000 rpm. Following centrifugation at 15 000 rcf for 5 min at 4 °C using an Eppendorf Centrifuge 5810R, 400 μL of supernatant was collected. The residue was then subjected to a second extraction using the same procedure. The combined supernatants were dried using EYELA CVE-200D Centrifugal Evaporator (TOKYO RIKAKIKAI CO., Tokyo,JP) and reconstituted with 200 μL of methanol containing internal standards (Aripiprazole-d8 at 200 ppb and Dehydroaripiprazole-d8 at 200 ppb). After 15 min of sonication and centrifugation at 15 000 rcf for 5 min at 4 °C, the supernatant was filtered through a 0.2-μm Minisart RC 4 filter (Sartorius Stedim Biotech GmbH, Goettingen, Germany) and subjected to LC–MS/MS.

#### LC–MS/MS conditions

Aripiprazole and Dehydroaripiprazole were analyzed using an Agilent 1290 UHPLC system coupled with an Agilent 6460 QQQ system (Agilent Technologies, Santa Clara, CA, USA). Two microliters of sample extract were injected into an ACQUITY UPLC HSS T3 column (2.1 × 100 mm, 1.8 μm) (Waters, Milford, MA, USA). The analytical column was maintained at 40 °C. The mobile phase compositions were (A) water with 10 mm ammonium acetate and 0.1% formic acid and (B) 90:10 (v/v) acetonitrile:water with 10 mm ammonium acetate and 0.1% formic acid. The flow rate was set to 0.3 mL/min. A gradient elution was applied: 0–2 min, 30–95% B; 2–2.5 min, 95% B; 2.5–6.0 min, 95–30% B. Sample ionization was achieved using a Jet Stream Technology Ion Source (AJS). Electrospray ionization in positive ion modes was used with the following parameters: 325 °C gas temperature, 7 L min^−1^ gas flow, 45 psi nebulizer, 325 °C sheath gas temperature, 11 L min^−1^ sheath gas flow, 3500 V capillary voltage, and 500 V nozzle voltage. MS acquisition was conducted in multiple reaction monitoring mode, with the transitions detailed in online Supplementary Table S1.

#### Calibration curve establishment

The calibration curve was prepared by spiking standard solutions with a series of concentrations into blank plasma, with concentrations ranging from 1 to 250 ng/mL for Aripiprazole and 0.5–100 ng/mL for Dehydroaripiprazole. The calibration curve was plotted using a linear regression of the area ratio of the analyte to the internal standard *v.* the target analyte concentration. The coefficient of determination (*r*^2^) was also calculated.

### Outcome measurement

Psychopathological measurements were conducted using the Mandarin versions of the PANSS (Cheng, Ho, Chang, Lane, & Hwu, 1996), Clinical Global Impression of Severity (CGI-S), and Personal and Social Performance Scale (PSP) (Wu et al., 2013). A trained psychiatrist performed these assessments at baseline and end of the 2-year follow-up period. Additionally, the participants completed a medication satisfaction questionnaire (MSQ) (Vernon et al., 2010) using a 7-point Likert scale to evaluate their subjective experience of dose reduction. Quality of life was assessed using the EuroQoL-5D visual analog scale (EQ-5D-VAS) (Chang et al., 2013) at baseline and after a 2-year follow-up. We also collected demographic data, clinical diagnoses, age at onset, illness duration, and employment status from the medical records and verified them through interviews conducted by a research assistant.

### Statistical analyses

To evaluate the differences in baseline demographics and psychopathology among the GDR, MT1, and MT2 groups, categorical variables were compared using chi-square tests, and continuous variables were examined using analysis of variance (ANOVAs). When the expected frequencies were < 5, the Fisher's exact test was used to identify significant differences. For variables that displayed significant differences in the ANOVA test, a post hoc analysis was conducted using Tukey's HSD test. The confidence intervals were two-sided and the statistical significance level was set at 5%. The effect size for datasets analyzed using ANOVA is represented by Eta-squared (*η*^2^), while for datasets analyzed using Fisher's exact test, it is defined by the odds ratio. All data analyses were performed using R Studio version 4.3.0.

Spearman correlation analysis was used to investigate the association between dosage and serum drug levels as well as the relationship between serum aripiprazole and dehydroaripiprazole levels. Additionally, simple linear regression models were employed to predict plasma drug levels based on dosage.

To explore whether maintaining a low serum drug level could still sustain the remitted state of schizophrenia, we categorized the participants into two groups based on their serum aripiprazole levels at the 2-year follow-up: those with aripiprazole levels ⩾120 ng/mL (high-level group) and those with levels <120 ng/mL (low-level group). Demographic data and clinical outcomes were compared using the chi-square test, Fisher's exact test, and ANOVAs, as described previously.

## Results

### Demographics and clinical characteristics

**Figure 1.**
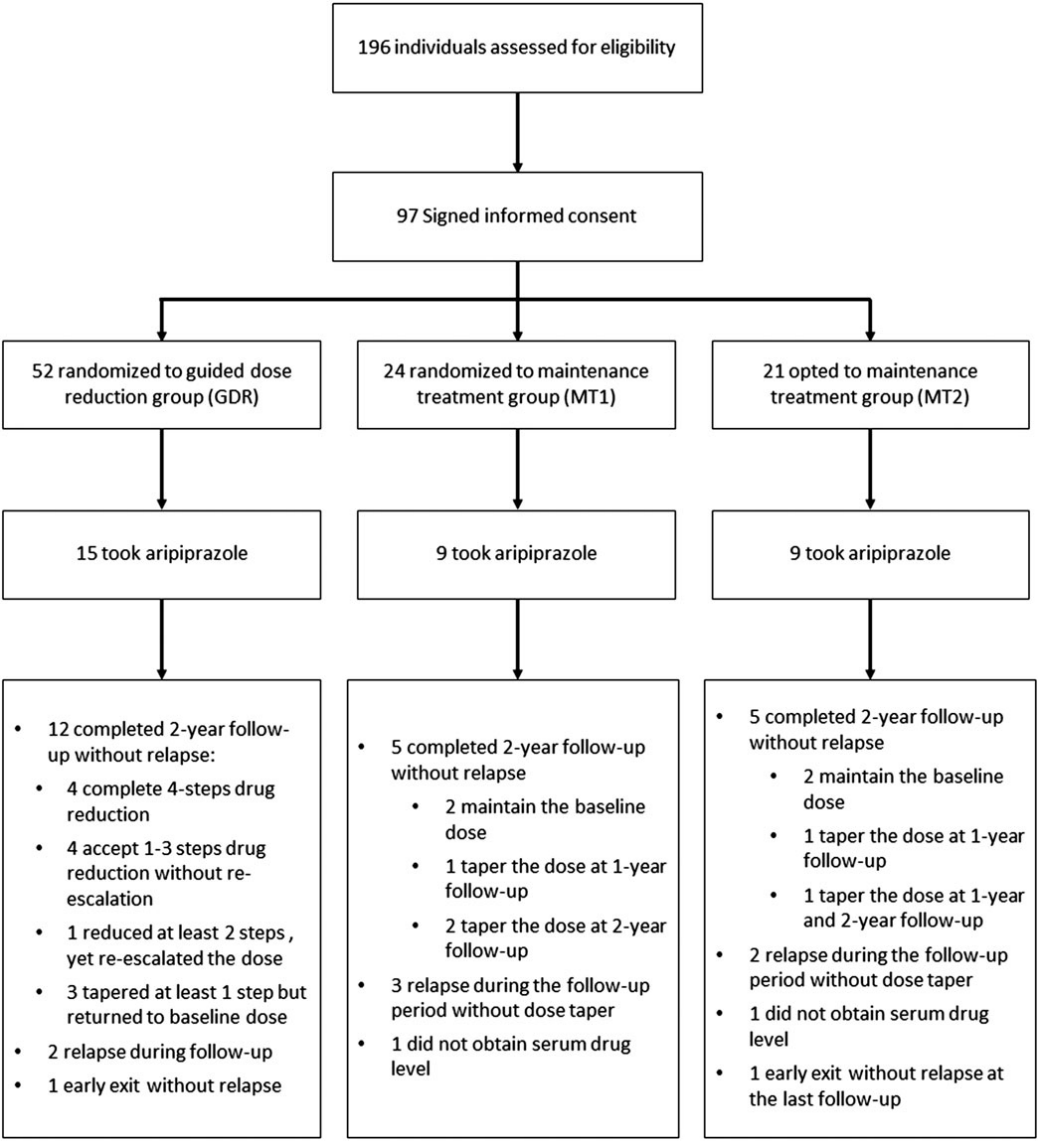
Flow diagram focused on patients treated with aripiprazole.

Among the 196 patients assessed for eligibility, 97 participated in the study, and 33 were treated with aripiprazole to examine their serum drug levels, including 15 patients in the GDR group, 9 in the MT1 group, and 9 in the MT2 group. The CONSORT flow diagram is shown in Fig. 1. The baseline characteristics are summarized in Table 1. No statistically significant differences in baseline characteristics were observed among the three groups, except that the GDR group had a shorter illness duration than the MT1 group.

**Table 1.** Baseline characteristics and number of relapses during the 2-year follow-up in the GDR, MT1, and MT2 groups

	GDR (*n* = 15)	MT1 (*n* = 9)	MT2 (*n* = 9)	Total (*n* = 33)	*p* value
Age, years (s.d.)	31.33 (7.72)	35.78 (7.08)	33.67 (8.57)	33.18 (7.78)	0.174
Sex, male (%)	8 (53.33%)	1 (11.11%)	3 (33.33%)	12 (36.36%)	0.112
Employment					0.227
Full-time	8	5	7	20	
Part-time	6	2	0	8	
No	1	2	2	5	
Age of onset (s.d.)	25.20 (6.42)	24.22 (6.42)	23.22 (5.78)	24.39 (6.11)	0.649
History of admission, no. (%)	5 (33.33%)	5 (55.56%)	1 (11.11%)	11 (33.33%)	0.135
History of relapse, no. (%)	11 (73.33%)	7 (77.78%)	7 (77.78%)	25 (75.76%)	0.957
Duration of illness (s.d.)	6.13 (3.81)	11.33 (5.89)	10.44 (5.81)	8.73 (5.42)	0.014^a^
Schizophrenia diagnosis no. (%)	9 (60%)	6 (66.67%)	7 (77.78%)	22 (66.67%)	0.670
PANSS total score (s.d.)	42.47 (13.68)	43.11 (6.43)	39.44 (9.03)	41.82 (10.72)	0.971
CGI-S score (s.d.)	1.87 (0.83)	1.67 (0.50)	1.78 (0.97)	1.79 (0.78)	0.552
PSP score (s.d.)	81.00 (9.89)	82.00 (5.98)	83.56 (8.22)	81.97 (8.35)	0.717
EQ-5D-VAS (s.d.)	76.47 (14.79)	73.89 (11.40)	76.67 (15.41)	75.82 (13.75)	0.687
MSQ score (s.d.)	5.60 (0.91)	5.56 (0.73)	5.89 (1.05)	5.67 (0.89)	1.000
Aripiprazole dose mg (s.d.)	9.93 (5.64)	12.78 (5.79)	7.64 (4.78)	9.03 (5.36)	0.333
Serum aripiprazole level mg/dL (s.d.)^a^	146.92 (124.38)	235.40 (100.94)	126.39 (69.07)	164.45 (112.22)	0.111
Serum dehyroaripiprazole level mg/dL (s.d.)^a^	55.05 (39.13)	84.17 (45.23)	35.83 (27.06)	57.60 (40.99)	0.191
Relapse (%)	2 (13.33%)	3 (33.33%)	2 (22.22%)	7 (21.21%)	0.508

Abbreviations: Adm, admission; CGI-S, Clinical Global Impression-Severity; EQ-5D-VAS, EuroQoL-5D visual analog scale; GDR, patients randomized to the guided dose reduction group; Hx, History; MSQ, medication satisfaction questionnaire; MT1, patients randomized to the maintenance group; MT2, patients volunteered to be the naturalistic observational group; PANSS, positive and negative syndrome scale; PSP, personal and social performance; s.d., standard deviation.

a Serum level data for one participant each in the MT1 and MT2 groups were not obtained. The mean and standard deviation (s.d.) of serum drug levels were calculated based on data from the remaining participants.

By the end of the 2-year follow-up period, excluding 2, 3, and 2 patients who experienced relapse in the GDR, MT1, and MT2 groups, respectively, no significant differences were detected in drug dosage, serum drug levels, and clinical psychopathology ratings among the three groups (Table 2).

**Table 2.** Comparison of clinical characteristics, drug dosage, and serum drug levels between groups after a 2-year follow-up^a^

	GDR (*n* = 13)	MT1 (*n* = 6)	MT2 (*n* = 7)	Total (*n* = 26)	*p* value	Eta-squared (*η*2)
PANSS total score (s.d.)	37.31 (8.75)	39.33 (4.59)	35.86 (8.03)	37.38 (7.62)	0.696	0.65%
CGI-S score (s.d.)	1.54 (0.66)	1.67 (0.52)	1.43 (0.79)	1.54 (0.65)	0.780	0.33%
PSP score (s.d.)	85.08 (6.92)	84.00 (5.25)	87.71 (4.35)	85.54 (5.92)	0.898	0.07%
EQ-5D-VAS (s.d.)	80.31 (12.44)	82.17 (13.57)	85.00 (10.41)	82.00 (11.88)	0.649	0.88%
MSQ score (s.d.)	5.54 (0.78)	5.83 (0.75)	6.00 (0.82)	5.73 (0.78)	0.343	3.75%
Aripiprazole dose mg (s.d.)	7.54 (6.07)	11.15 (2.57)	5.98 (3.30)	7.95 (5.02)	0.246	5.57%
Serum aripiprazole level mg/dL (s.d.)^b^	111.87 (121.08)	186.17 (22.18)	93.03 (75.92)	122.58 (100.8)	0.254	5.87%
Serum dehyroaripiprazole level mg/dL (s.d.)^b^	40.43 (38.46)	76.07 (24.37)	23.29 (19.21)	43.57 (36.02)	0.154	9.03%
Aripiprazole *C*/*D* ratio (s.d.)^b^	14.82 (9.69)	16.70 (3.53)	15.55 (8.39)	15.98 (8.15)	0.997	<0.01%
Dehydroaripiprazole *C*/*D* ratio (s.d.)^b^	5.36 (3.55)	6.82 (1.44)	3.89 (2.31)	5.59 (2.96)	0.982	<0.01%

Abbreviations: CGI-S, Clinical Global Impression-Severity; EQ-5D-VAS, EuroQoL-5D visual analog scale; GDR, patients randomized to the guided dose reduction group; Hx, History; MSQ, medication satisfaction questionnaire; MT1, patients randomized to the maintenance group; MT2, patients volunteered to be the naturalistic observational group; PANSS: positive and negative syndrome scale; PSP: personal and social performance; s.d.: standard deviation.

a Follow-up data were calculated using the Last Observation Carried Forward (LOCF) method. All patients who experienced relapse during the follow-up period were excluded from the analysis.

b Serum level data for one participant in the MT1 group and one in the MT2 group were not obtained. The mean and standard deviation (s.d.) of serum drug levels were calculated based on data from the remaining participants.

### Composition of blood samples

As shown in Fig. 1, based on the nomenclature of our previous report regarding the GDR group (Liu et al., 2023a), four sequential reducers successfully completed four steps of dose reduction: four modest reducers tapered only 1 to 3 steps over 2 years, one alert reducer reduced 2 steps but then partially re-escalated to a dose below the baseline level, three baseline returners initially reduced the dosage but subsequently resumed to their baseline levels, two failed reducers experienced relapses during the tapering process, and one participant withdrew from the trial after undergoing only 1-step tapering. Together with three patients in the MT1 group and two in the MT2 group who changed dosage during the course on their own accord, a total of 126 blood samples were collected from 31 patients who were receiving varying doses of aripiprazole at different timepoints, including 89 samples from GDR patients at 47 different doses and 37 samples from MT1 and MT2 patients at 22 different doses.

### Correlations between serum aripiprazole and dehydroaripiprazole concentrations

The overall average baseline aripiprazole dose was 9.03 mg, with corresponding serum levels of 164.45 mg/dL for aripiprazole and 57.60 mg/dL for dehydroaripiprazole. The correlation coefficient between plasma aripiprazole and dehydroaripiprazole levels was 0.923 (*p* < 0.0001). A linear regression model illustrating the relationship between aripiprazole and dehydroaripiprazole serum levels is shown in online Supplementary Fig. S1.

### Correlations between dose and serum drug levels

In the scatterplots (online Supplementary Fig. S2), we identified 16 samples with apparently low plasma drug concentration-to-dose ratios, confirmed if patients adhered to the prescribed medication doses or not, and excluded 13 samples from those who confessed non-adherence from further analysis. In the remaining 113 data points, the correlation coefficients between dosage and plasma aripiprazole level and between dosage and dehydroaripiprazole level were 0.853 (*p* < 0.0001) and 0.864 (*p* < 0.0001), respectively (online Supplementary Fig. S3).

Upon maintaining a stable dosage over a period of months, the average concentration-to-dose (*C*/*D*) ratios ranged from 3.68 to 34.53 ng/mL/mg (mean: 17.79 ng/mL/mg) for concentration of aripiprazole and 0.69 to 13.13 ng/mL/mg (mean: 6.27 ng/mL/mg) for concentration of dehydroaripiprazole. The plasma *C*/*D* ratios observed in our study were compared with those reported in previous literature (Citrome, Josiassen, Bark, Salazar, & Mallikaarjun, 2005; Jönsson, Spigset, & Reis, 2019; Kim et al., 2008; Kirschbaum et al., 2008; Lin, Chen, & Liu, 2011; Molden, Lunde, Lunder, & Refsum, 2006; Nakamura, Mihara, Nagai, Suzuki, & Kondo, 2009; Rafaniello et al., 2018) (online Supplementary Table S2), closer to Eastern populations and, in general, higher than those in Western populations.

### Constellations of individual trajectories of dose and serum drug concentration

**Figure 2.**
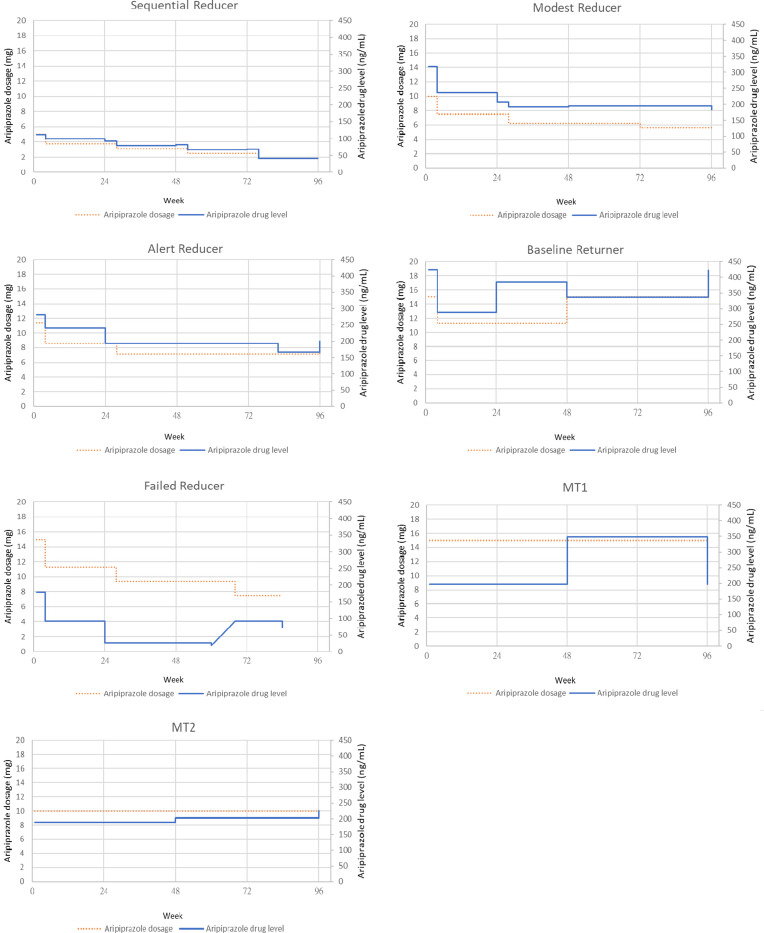
Case illustration of dose variation and corresponding drug level. MT1, patient randomized to maintenance group; MT2, patient volunteered to be the naturalistic observational group.

To further elucidate the close relationship between dose and serum drug level, we created run charts displaying the various doses and their corresponding drug levels in patients with different dose-tapering trajectories. These selective cases illustrate the highly correlated parallel relationships between the dose and drug concentrations, as shown in Fig. 2.

### Comparisons between patients with low- and high- serum drug levels

Table 3 presents comparisons of the clinical characteristics and outcomes between patients under high- and low- serum drug level, cutoff by 120 ng/mL at baseline. The high drug level group exhibited a significantly higher frequency with a history of admission and higher baseline aripiprazole dosage compared to the low drug level group. Conversely, no statistically significant differences were observed pertaining to psychopathology, history of relapse, personal social functioning, and self-rated quality of life between groups.

**Table 3.** Comparison of the clinical characteristics of patients grouped by serum aripiprazole levels at the end of follow-up^a^

	Serum aripiprazole level >120 ng/ml (*n* = 16)	Serum aripiprazole level <120 ng/ml (*n* = 15)	Total (*n* = 31)	*p* value	Effect size^b^
Age, years (s.d.)	34.25 (6.34)	30.87 (8.71)	32.61 (7.64)	0.224	
Sex, male (%)	3 (18.75%)	9 (60%)	12 (38.71%)	0.029^a^	
Employment				0.694	
Full-time	8	10	18		
Part-time	5	3	8		
No	3	2	5		
Onset age (s.d.)	24.44 (6,21)	24.20 (6.60)	24.32 (6.29)	0.918	
Hx of adm no. (%)	9 (56.25%)	2 (13.33%)	11 (35.48%)	0.023^a^	
Hx of relapse no. (%)	12 (75%)	11 (73.33%)	23 (74.19%)	0.916	
Duration of illness (s.d.)	9.69 (4.42)	6.67 (5.39)	8.23 (5.07)	0.098	
Schizophrenia diagnosis no. (%)	13 (81.25%)	9 (60%)	22 (70.97%)	0.252	
BL APZ dose mg (s.d.)	12.90 (4.51)	7.50 (5.75)	10.29 (5.75)	0.007^b^	
BL APZ level mg/dL (s.d.)	244.13 (89.08)	79.46 (59.06)	164.45 (112.22)	<0.001^b^	
BL PANSS total score (s.d.)	41.75 (7.47)	42.87 (13.90)	42.29 (10.88)	0.781	
BL CGI-S score (s.d.)	1.94 (0.68)	1.73 (0.88)	1.84 (0.78)	0.475	
BL PSP score (s.d.)	81.50 (7.28)	81.80 (9.91)	81.65 (8.50)	0.924	
BL EQ-5D-VAS (s.d.)	71.56 (14.23)	79.13 (11.76)	75.23 (13.44)	0.119	
BL MSQ score (s.d.)	5.69 (0.79)	5.47 (0.92)	5.58 (0.85)	0.478	
Relapse no. (%)	4 (25%)	3 (20%)	7 (22.58%)	0.739	1.31
F/U APZ dose mg (s.d.)	10.97 (4.37)	5.09 (4.29)	8.12 (5.20)	<0.001^b^	32.94%
F/U APZ level mg/dL (s.d.)	202.91 (76.35)	48.73 (35.69)	128.31 (98.20)	<0.001^b^	63.61%
F/U PANSS total score (s.d.)	40.69 (9.54)	40.20 (10.66)	40.45 (9.93)	0.894	0.06%
F/U CGI-S score (s.d.)	1.88 (0.81)	1.67 (0.82)	1.77 (0.80)	0.481	1.73%
F/U PSP score (s.d.)	82.06 (8.44)	85.00 (6.47)	84.75 (7.16)	0.288	3.88%
F/U EQ-5D-VAS (s.d.)	78.31 (15.82)	80.93 (11.79)	79.58 (13.85)	0.607	0.92%
F/U MSQ score (s.d.)	5.81 (0.75)	5.40 (0.74)	5.61 (0.76)	0.134	7.59%

Abbreviations: Adm, admission; APZ, aripiprazole; BL, baseline; CGI-S, Clinical Global Impression-Severity; EQ-5D-VAS, EuroQoL-5D visual analog scale; F/U, 2-year follow-up; Hx, History; MSQ, medication satisfaction questionnaire; PANSS, positive and negative syndrome scale; PSP, personal and social performance; s.d., standard deviation.

a Follow-up data were calculated using the Last Observation Carried Forward (LOCF) method. We used the last available data before relapse for patients who experienced relapse.

b The effect size for datasets analyzed using ANOVA is represented by Eta-squared (*η*^2^), while for datasets analyzed using Fisher's exact test, it is defined by the odds ratio.

At baseline, there were 19 and 12 participants in the high- and low-level groups, respectively. Three participants transitioned from the high-level to the low-level group by the end of the study. Two of them were from the GDR group, yet both tapered their dosage faster than the designated tempo; one experienced a relapse and the other was maintained in remission. The third patient was from the maintenance group (at the same dose [7.5 mg/d] throughout the course) and her serum drug level varied from 122.76 ng/mL to 110.82 ng/mL. By the end of the 2-year follow-up, 80% (12/15) of the patients were able to sustain remission, even with notably low plasma aripiprazole levels (< 120 ng/mL).

## Discussion

In our earlier investigation, the GARMED trial, we noted that half of our patients could maintain remission at dosages below the previously defined lowest effective dose, the chlorpromazine equivalent (CPZE) 200 mg/d (Liu et al., 2023a, 2023b), and some successfully reduced a substantial proportion of their baseline doses during a 2-year exponential dose tapering without relapse. These findings challenge the current knowledge regarding the increased risk of relapse if the dose is reduced to below CPZE 150 or 200 mg/d (Leucht et al., 2021; Tani et al., 2020). While the doses at each time point in the GARMED trial are based on the agreement between patients and doctors via shared decision-making, we need to confirm whether patients took the designated dosage during a certain period. Subsequently, we delved into observations based on the serum drug levels of a subset of patients treated with aripiprazole and revealed similar findings; patients could maintain remission at a level lower than the previously assumed limit of 120 ng/mL (Hart et al., 2022). As this is one of the few studies focusing on serum antipsychotic drug levels during dose tapering in the maintenance phase, several important points are worthy of further discussion.

First, the positive relationship illustrated by the high correlations between aripiprazole dosage and serum drug levels in the scatter plots (online Supplementary Figs S2 and S3) suggests that most participants adhered to the dose reduction protocol. This correlation was exemplified in selected case illustrations (Fig. 2) in which the serum drug level correlated well with the administered dosage. This relationship was consistent among the three groups, as shown by the comparable *C*/*D* ratios (Table 2). Moreover, a small yet significant number of outliers confessed that they did not adhere to the dose reduction protocol, a trend that was easily identified in the scatter plots (online Supplementary Fig. S2). Using TDM as a viable strategy for validating adherence (Pennazio, Brasso, Villari, & Rocca, 2022), these observations verified the success and practicality of our exponential dose reduction algorithm.

Second, recent dose reduction trials on stable patients with psychosis either reported an equivalent (Huhn et al., 2021) or a doubled risk of relapse (Moncrieff et al., 2023) compared to the maintenance group; neither trial validated the patients' medication adherence by TDM. Therefore, the question arises that what if patients who experienced relapses were those who tapered down the dose too fast and/or too much at a time, rendering an inflated rate of relapse on their way to discontinuing antipsychotics? In our cases, one patient's TDM trajectory was visibly not parallel to the reported doses. Upon confirmation, he confessed that he often skipped doses when feeling well, yet resumed some doses when feeling unwell (failed reducer in Fig. 2). Because the abrupt discontinuation of antipsychotics potentially leads to dopamine supersensitivity psychosis (Chouinard et al., 2017; Moncrieff, 2015), a patient's understanding and motivation to cooperate during the tapering process are important factors for successful dose reduction.

Third, in our trial, comparisons of clinical features between the high- and low drug level groups revealed no significant differences, including no higher relapse rates in patients treated with a drug concentration below the suggested therapeutic reference range. Correspondingly, the therapeutic dose required for patients in the remitted state may be considerably lower than that required for patients in the acute psychotic state. However, the mean *C*/*D* ratios for both aripiprazole and dehydroaripiprazole in our samples were notably higher than those in studies involving Caucasians, and comparable to those of other Asian studies (online Supplementary Table S2). This finding reminds us that the doses reported in our GARMED trial might not be directly applicable to the trials conducted in Caucasian populations, as our patients could reach a relatively high serum drug concentration with the same aripiprazole dose than Caucasians.

Fourth, there was no significant difference in the quality of life and subjective satisfaction between the low- and high drug level groups, failing to support the previous assumption that dose reduction improved the potential attenuation of the burden related to antipsychotics (Dubath et al., 2021; Sabé et al., 2023). Indeed, an in-depth analysis of dose-tapering trajectories in our trial revealed that only a subset of patients who could successfully taper four times and reduce more than half of their baseline doses endorsed better functioning and subjective well-being (Liu et al., 2023a). The small sample size available for TDM makes it difficult to verify this finding. Further investigations are warranted to determine the minimal effective dose and minimum serum drug level for remitted psychosis.

### Limitations

Our study has several limitations. First, the doses of our samples from a highly selected group of stable patients were skewed to a lower level, with the majority not exceeding 15 mg/d. Thus, the extrapolation of our findings to patients not under a stable course or treated with higher doses should be cautious and require more data from higher dose ranges. Second, patient genotypes of CYP2D6 and CYP3A4 as well as co-administered drugs that might induce or inhibit these enzymes were not evaluated in this study, while both factors might affect the blood concentration of aripiprazole and dehydroaripiprazole. Third, the timing of blood sample collection was not always at the trough, as some patients preferred to take medication in the morning and others at bedtime. Because the half-life of aripiprazole is long, we assumed that a relatively stable drug level had been achieved when we measured it at least 4 weeks under the same dosing schedule, and the blood sample was collected at least 12 h apart from the latest dosing. Fourth, we did not collect blood samples immediately after the patient had a relapse, as the priority at that moment was to resume higher doses of antipsychotics. Since the interval between the last observation and the current status might be too long, the drug concentration measured at the last observation would not be informative for predicting indexed relapse. Lastly, our participants received a wide variety of antipsychotic treatments, and we did not have sufficiently large subsamples treated with other antipsychotics to retest what we conducted on aripiprazole and see if patients could regain better functioning and quality of life under lower serum drug levels of antipsychotics with less dopamine partial agonistic properties.

## Conclusions

In summary, this study provides evidence by TDM to verify our findings from an exponential dose reduction trial. Most patients, either those undergoing dose tapering or their maintenance counterparts, demonstrated stability during the 2-year follow-up, with approximately 40% of them maintaining a serum aripiprazole concentration below 120 ng/mL. Not only the dose but also the therapeutic serum drug level required for effective prophylaxis of relapse may be lower than those previously assumed.

## Supporting information

Liu et al. supplementary material Liu et al. supplementary material

## Supplementary material

The supplementary material for this article can be found at https://doi.org/10.1017/S0033291724002356
