# Supplementary material for: Verification of successful maintenance by serum drug level during a guided antipsychotic reduction to reach minimum effective dose (GARMED) trial
Source: Psychol Med. 2024 Sep 26;54(14):3906–16. doi: 10.1017/S0033291724002356 (PMC11578910; doi:10.1017/S0033291724002356)
Supplement: Liu et al. supplementary material [file S0033291724002356sup001.doc]

Supplement Table 1. Mass parameters and MRM transitions for Aripiprazole and Dehydroaripiprazole

| Analytes | MRM Transition | | Function |
| --- | --- | --- | --- |
| Aripiprazole | 448.2🡪285.1 | Quantification | |
|  | 448.2🡪98.1 | Qualification | |
| Aripiprazole -d8 | 456.2🡪293.1 | Quantification | |
|  | 456.2🡪106.1 | Qualification | |
| Dehydroaripiprazole | 446.1🡪285.1 | Quantification | |
|  | 446.1🡪98.1 | Qualification | |
| Dehydroaripiprazole -d8 | 454.2🡪293.1 | Quantification | |
|  | 454.2🡪106.1 | Qualification | |

Supplement Table 2. Comparison of plasma concentration-dose ratios between our study and previous studies

|  | **C/D of ARI** | **C/D of DARI** | **C/D of ARI+DARI** |
| --- | --- | --- | --- |
| Our Study | 17.79±7.23 ng/mL/mg | 6.27±2.09 ng/mL/mg | 24.07±8.70 ng/ml/mg |
| Rafaniello 2018 | 16.41±10.26 ng/mL/mg |  |  |
| Molden 2006 | 11.57 ng/mL/mg | 4.02 ng/mL/mg | 15.59 ng/ml/mg |
| Jönsson 2019 | 11.99 ng/mL/mg |  |  |
| Lin 2011 | 14.80±6.00 ng/mL/mg | 6.10±2.40 ng/mL/mg |  |
| Kim 2008 | 16.50±7.00 ng/mL/mg | 5.30±1.90 ng/mL/mg |  |
| Nakamura 2009 | 12.60±4.90 ng/mL/mg | 5.70±1.90 ng/mL/mg |  |
| Kirschbaum 2008 | 11.40±7.30 ng/mL/mg | 4.00±4.00 ng/mL/mg |  |
| Citrome 2005 | 7.90±2.70 ng/mL/mg | 3.40±0.90 ng/mL/mg |  |

Abbreviations: C/D, Concentration-to-dose ratio; ARI, aripiprazole; DARI, dehydroaripiprazole


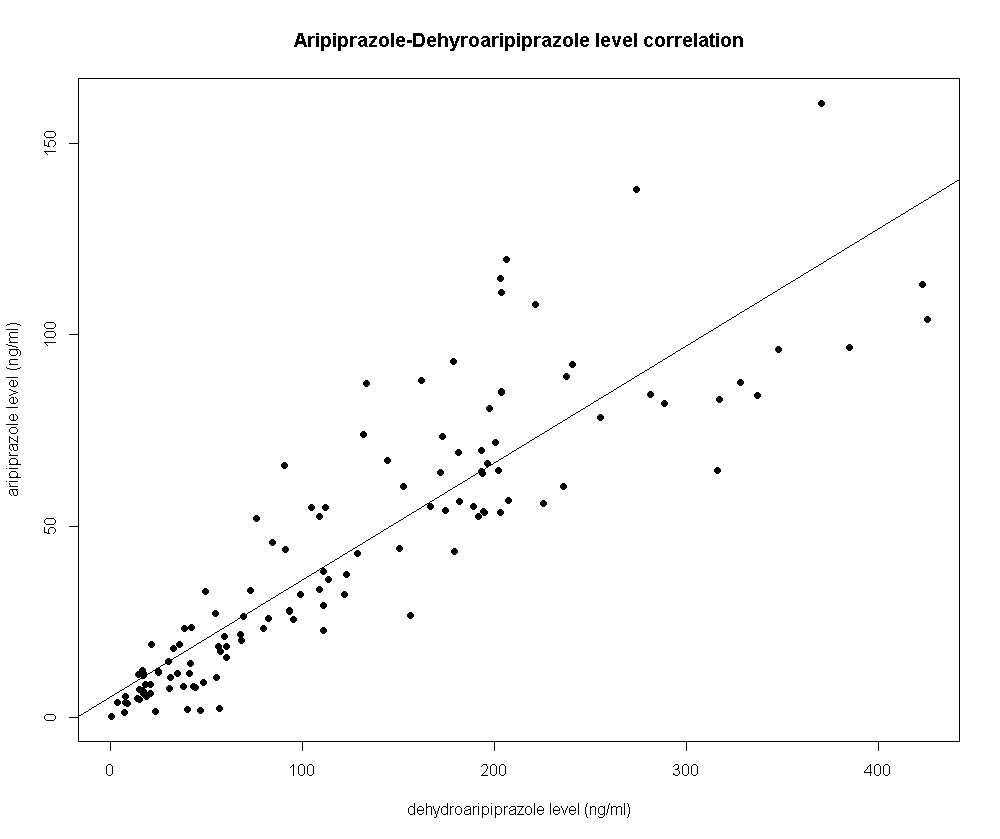


Supplement Figure 1. Scatter plot of dehydroaripiprazole/aripiprazole serum drug levels with regression lines


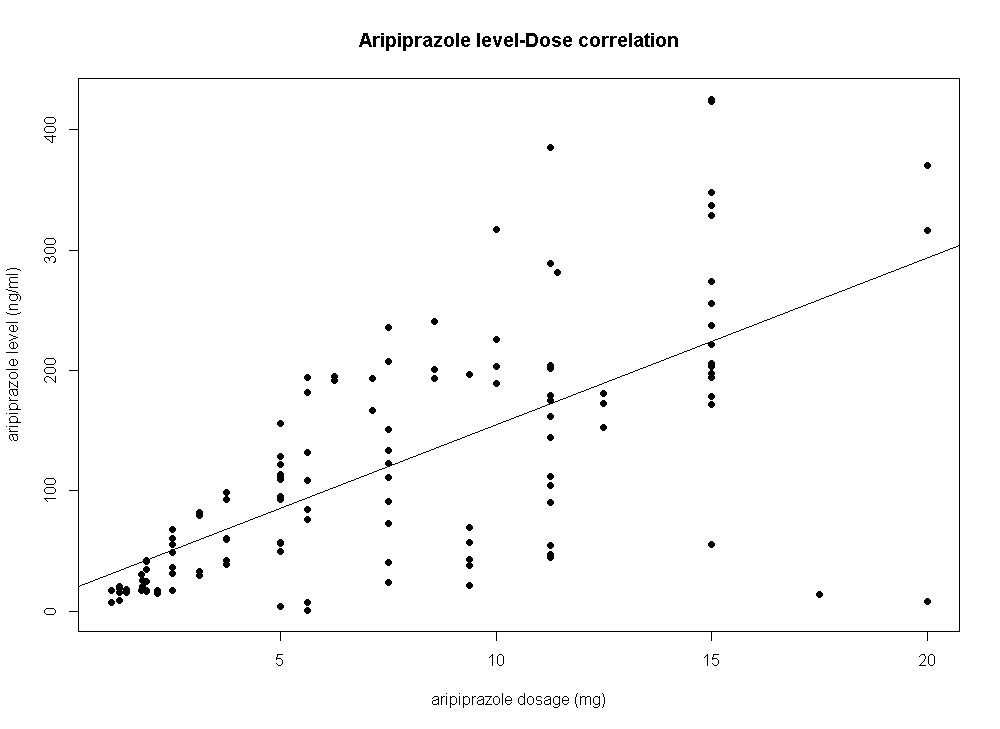

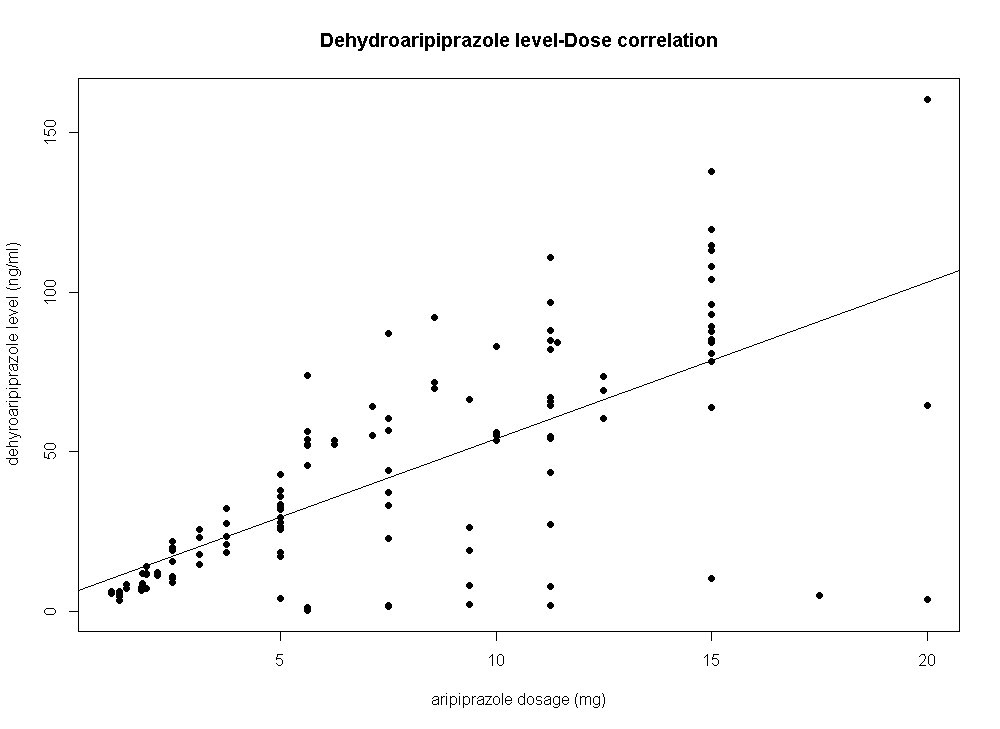


Supplement Figure 2: Left: Scatter plot of aripiprazole dose and serum aripiprazole concentration with regression line before exclusion of outliers; Right: Scatter plot of aripiprazole dose and serum dehydroaripiprazole concentration with regression line before exclusion of outliers


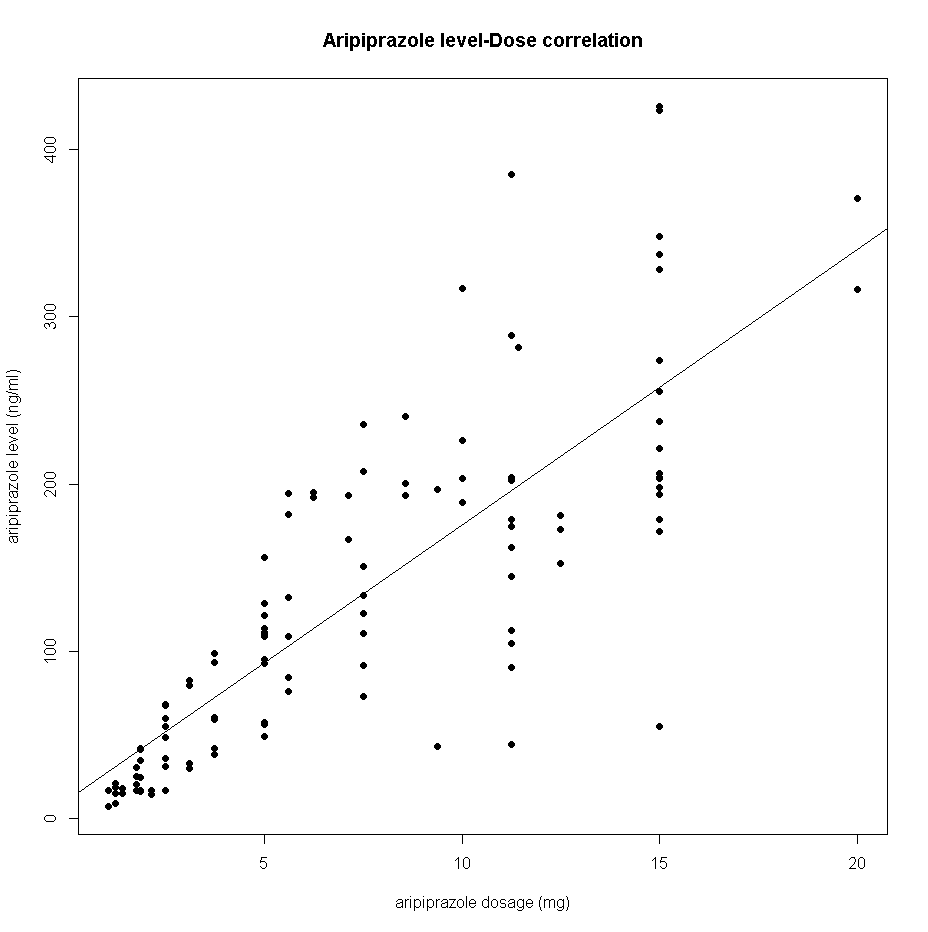

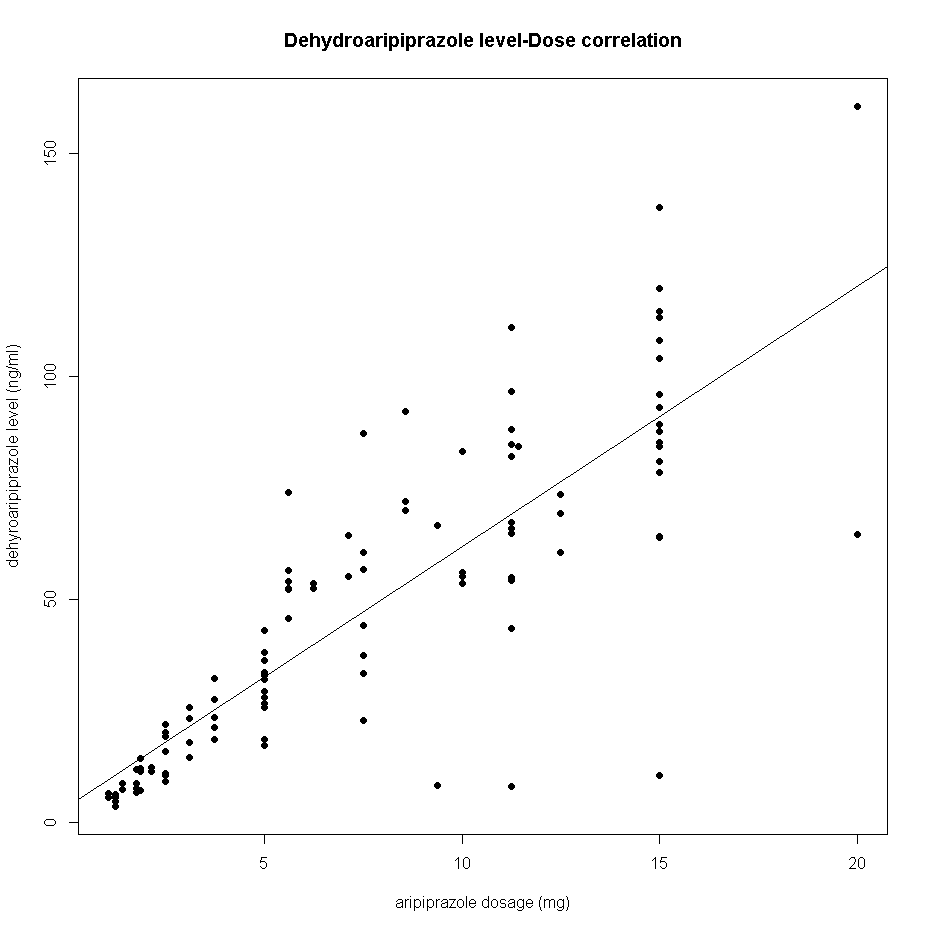


Supplement Figure 3. Scatter plots. Left: Scatter plot of aripiprazole dose and serum aripiprazole concentration with a regression line; Right: Scatter plot of aripiprazole dose and serum dehydroaripiprazole concentration with a regression line
